# Draft Genome Sequence of “*Candidatus* Liberibacter asiaticus” Strain GZQL4, from Guizhou, China

**DOI:** 10.1128/mra.00377-23

**Published:** 2023-06-29

**Authors:** Kehong Liu, Ziqin Bai, Xuejin Cui, Qian Lin

**Affiliations:** a National Citrus Engineering Research Center, Citrus Research Institute, Southwest University, Chongqing, China; b Fruit Research Institute, Guizhou Provincial Academy of Agricultural Sciences, Guiyang, Guizhou, China; The University of Arizona

## Abstract

Here, we announce the draft genome sequence of “*Candidatus* Liberibacter asiaticus” strain GZQL4, which was collected from Guizhou, China. The GZQL4 strain has a genome size of 1,234,029 bp, a G+C content of 36.5%, 1,204 predicted open reading frames, and 53 RNA genes.

## ANNOUNCEMENT

The bacterium “*Candidatus* Liberibacter asiaticus,” which is associated with huanglongbing (HLB), is a yet uncultured alphaproteobacterium (1). HLB is a threatening disease of citrus worldwide and has caused enormous economic losses (2). HLB was first discovered in Chaozhou, Guangdong, China, in 1919 (3). HLB was found only in Guangdong, Guangxi, Fujian, and Taiwan before the 1970s and then gradually spread in China (4). As of 2018, HLB has been detected in over 330 counties of 11 provinces of China (5). “*Ca*. Liberibacter asiaticus” has not yet been cultured *in vitro*, which presents challenges for researching the bacterium using traditional microbial research methods. Genome sequencing data can provide important information for understanding basic molecular and evolutionary characteristics. Here, we report a draft genome sequence of “*Ca*. Liberibacter asiaticus” from Guizhou, China.

“*Ca*. Liberibacter asiaticus” strain GZQL4 was originally collected from an HLB-infected navel orange (Citrus sinensis L. Osbeck) in Qinglong County (25.7692N, 105.2977E), Guizhou Province, China. Total DNA was extracted from citrus pith using the Biospin Omni plant genomic DNA extraction kit (BioFlux, China). The concentration of “*Ca*. Liberibacter asiaticus” was determined with real-time PCR, which yielded a cycle threshold (*C_T_*) value of 20.67 (6). The DNA was amplified using the GenomiPhi v2 DNA amplification kit (GE Healthcare, Chalfont St. Giles, UK) and then was sequenced by Novogene on the NovaSeq platform (150-bp paired-end reads; Illumina Inc., San Diego, CA, USA). Quality control was performed using FastQC (https://www.bioinformatics.babraham.ac.uk/projects/fastqc), and a total of 6.09 × 10^7^ clean reads, with a read length of 150 bp, were generated by the NovaSeq sequencing. The clean reads were mapped against “*Ca*. Liberibacter asiaticus” strain A4 (7) and three prophages of “*Ca*. Liberibacter asiaticus” (SC1, SC2, and P-JXGC-3) (8, 9) using CLC Genomics Workbench v11.0.1 (Qiagen) with default settings, and a consensus sequence with 1,228,164 bp and 64× coverage was obtained. Citrus genome sequences were removed from the clean reads, using C. sinensis (10) as the reference, by Bowtie2 v2.2.5 with default settings (11), and *de novo* assembly of the filtered reads was performed using CLC Genomics Workbench v11.0.1, which generated 4,518 contigs, ranging from 916 bp to 57,897 bp. Contigs were used as queries in a BLAST search against the consensus sequence with BLAST+ v2.9.0 (12), and the genome was assembled by combining the consensus sequence and the contigs. The final gaps were closed by Sanger sequencing of the PCR products across these gaps.

The draft genome of GZQL4 contains 1,234,029 bp, with a G+C content of 36.5%. The GZQL4 genome contains two contigs, i.e., a chromosome (1,194,518 bp) and a type I prophage (39,511 bp). Annotation was performed using the RAST server (http://rast.nmpdr.org) (13), which predicted 1,204 open reading frames and 53 RNA genes.

### Data availability.

The draft genome sequence and sequencing reads of “*Candidatus* Liberibacter asiaticus” strain have been deposited in GenBank under accession numbers CP124118 and CP124119. The versions described in this paper are the first versions.
